# Thermal Stability of *Type II* Modifications by IR Femtosecond Laser in Silica-based Glasses

**DOI:** 10.3390/s20030762

**Published:** 2020-01-30

**Authors:** Shu-En Wei, Yitao Wang, Heng Yao, Maxime Cavillon, Bertrand Poumellec, Gang-Ding Peng, Matthieu Lancry

**Affiliations:** 1 Photonics and Optical Communications Group, School of Electrical Engineering, University of New South Wales, Sydney, NSW 2052, Australiag.peng@unsw.edu.au (G.-D.P.); 2 Institut de Chimie Moléculaire et des Matériaux d’Orsay, Université Paris Saclay, 91400 Orsay, France; heng.yao22021992@gmail.com (H.Y.); maxime.cavillon@u-psud.fr (M.C.); bertrand.poumellec@u-psud.fr (B.P.)

**Keywords:** fiber Bragg gratings (FBGs), thermal stability, *Type II* modification, femtosecond laser, silica-based glasses

## Abstract

Femtosecond (fs) laser written fiber Bragg gratings (FBGs) are excellent candidates for ultra-high temperature (>800 °C) monitoring. More specifically, *Type II* modifications in silicate glass fibers, characterized by the formation of self-organized birefringent nanostructures, are known to exhibit remarkable thermal stability around 1000 °C for several hours. However, to date there is no clear understanding on how both laser writing parameters and glass composition impact the overall thermal stability of these fiber-based sensors. In this context, this work investigates thermal stability of *Type II* modifications in various conventional glass systems (including pure silica glasses with various Cl and OH contents, GeO_2_-SiO_2_ binary glasses, TiO_2_- and B_2_O_3_-doped commercial glasses) and with varying laser parameters (writing speed, pulse energy). In order to monitor thermal stability, isochronal annealing experiments (Δt⁓ 30 min, ΔT⁓ 50 °C) up to 1400 °C were performed on the irradiated samples, along with quantitative retardance measurements. Among the findings to highlight, it was established that ppm levels of Cl and OH can drastically reduce thermal stability (by about 200 °C in this study). Moreover, GeO_2_ doping up to 17 mole% only has a limited impact on thermal stability. Finally, the relationships between glass viscosity, dopants/impurities, and thermal stability, are discussed.

## 1. Introduction

Over the past 10 years, development of high temperature optical fiber-based sensors, such as fiber Bragg gratings (FBGs), has drawn great attention due to their wide range of applications, including temperature profiling of equipment in manufacturing industry like smelters or laser additive manufacturing [1], monitoring temperature and strain in oil, gas and geothermal industries [2], or again temperature and pressure monitoring in next generation sodium cooled nuclear reactors, airplanes engines, etc.

Conventional FBGs typically are inscribed into fibers composed of germanium-doped silica cores, due to their photosensitivity under intense ultraviolet (UV) light irradiation [3]. Under such irradiation, point defects are created inside the glass core, yielding changes in the glass macroscopic properties such as density and refractive index. These refractive index changes are positive with respect to the pristine (i.e., non-irradiated) glass and are often referred to as *Type I* index change [4]. When UV-induced refractive index changes are chosen as a way to inscribe photonic devices (i.e., *Type I* Bragg grating sensors), the later can only be used up to ~450 °C. Above this temperature, the imprinted object would progressively be thermally erased, making *Type I* Bragg grating sensors no longer suitable for temperature monitoring [5]. To go beyond the thermal limitations imposed by the technology described above, it is possible to imprint other kinds of modifications in the glass that can withstand higher temperature operation. One possibility is to make femtosecond *Type II* fiber Bragg gratings (also referred to *Type II* fs-FBGs) [5,6]. As opposed to *Type I* refractive index change, the *Type II* modification corresponds to the formation of nanostructures in most silica-based glasses associated with a high level of anisotropic index change [7]. It has been demonstrated that *Type II* fs-FBGs exhibited thermal stability at ~1000 °C for at least several hours in silica [8,9] and up to 150 h provided the FBGs have been stabilized [5,6].

Additionally, it is important to point out that both the structural characteristics of the fs-*Type II* modification and its thermal stability are both dependent on the fiber core glass composition. In fiber fabrication processes, germanium oxide (GeO_2_, typically up to 30 mole percent) is arguably the most common fiber core dopant and is used to increase the refractive index of the core relative to the one of the cladding material (being silica, SiO_2_) in order to form a guiding core [10]. In addition to GeO_2_, other common dopants such as fluorine (F), aluminum oxide (Al_2_O_3_), boron oxide (B_2_O_3_), and titanium dioxide (TiO_2_) also are incorporated into the fiber, and this for different purposes [11,12,13]. For example, we recently investigated “high temperature fibers” composed of aluminosilicate cores made by the molten core method [4]. For the Y_2_O_3_-Al_2_O_3_-SiO_2_ core composition, the measured birefringence does not decrease when tested up to 1000 °C for 30 min, while for the SiO_2_ F300 cladding it decreased by ~30%. These results suggest that inscription of “*Type II fs-IR*” modifications in such fibers could be employed to make FBGs with high thermal stability and to potentially overcome the current performances of pure silica core or Ge-doped SiO_2_ fibers. During the incorporation of dopants or along the fabrication process, impurities such as chlorine (Cl) and hydroxyl groups (OH) are also inevitably introduced [14,15]. All these intended incorporated elements or unintended contaminations would influence the formation of fs-*Type II* modifications and their thermal stability. To date, the relevant research on the impact of these incorporated elements/impurities on the formation of fs-*Type II* modification and thermal stability is sparse and not systematic [4,16,17,18,19,20].

In addition to the material, laser-writing process has been proved to be another important factor influencing the thermal performance of FBGs. It has been theoretically shown that thermal stability of FBGs can be enhanced by increasing the writing time [21], while the effect of pulse intensity and repetition rate on the thermal performance of FBGs has been investigated elsewhere (Reference [22]). However, to date there is no clear understanding on how both laser writing parameters and glass composition impact the overall thermal stability of these fiber-based sensors. Consequently, it prevents reliable lifetime and performance predictions of the fabricated objects, as the underlying mechanisms of erasure at high temperatures are not well identified.

It is not convenient to carry out a controlled and systematic research work by directly using fibers, because of their (usually) small core sizes along with the variety of their fabrication processes. In addition, to ensure a good quality of the irradiations and accuracy of measurements, homogeneous glass samples, rather than fiber samples, which have gradient distributed dopants concentration in their cores, are preferred in the present study. Consequently, in this work, the formation of fs-*Type II* modifications and their thermal stability in silica-based bulk glasses, instead of fibers, are investigated. More specifically, the impact of the writing conditions, especially writing speed, on fs-*Type II* modification thermal stability, is studied. Furthermore, we investigate the impact of impurities (Cl and OH) and dopants (GeO_2_, TiO_2_ and B_2_O_3_) on fs-*Type II* modification thermal stability. This work is expected to help in the future high temperature fiber sensor designs based on investigated oxide glass compositions. 

## 2. Materials and Methods

In order to investigate the impact of the laser writing speed on thermal stability of fs-*Type II* modifications, a standard grade fused silica sample from Heraeus (Suprasil CG^®^) was chosen.

To study the impact of composition on thermal stability, several sets of optical glasses are investigated. The samples are silica glasses with different Cl and OH concentrations in them (which are considered impurities as mentioned above). More specifically, we investigated Type I (Infrasil 301^®^), Type II (Homosil^®^), Type III (Suprasil CG^®^ and Spectrosil 2000^®^), and Type IV (Suprasil F300^®^) silica glasses. The second set of samples is composed of 4 germanosilicate (SiO_2_-GeO_2_) glasses with GeO_2_ concentration ranging from 1.9 to 16.9 mole%. These SiO_2_-GeO_2_ glasses, all with low OH content (<0.1 ppm) and high Cl content (typ. 2000 ppm), were manufactured by chemical vapor deposition (CVD) method. Finally, the last set of samples includes four silicate glasses doped with different oxide components (among which TiO_2_, GeO_2_, B_2_O_3_) and one fused silica glass for sake of comparison. More specifically, these samples are Infrasil 301^®^ for the silica one, two germanosilicate glasses, one TiO_2_-doped silica (ULE^®^, Corning), and one alumino-borosilicate glass (Schott Borofloat33^®^).

To help the reader, Table 1 summarizes the glass samples investigated in this study, along with their compositions, impurity types and concentrations, and annealing temperature (T_a_, defined as the temperature at which the viscosity is 10^13^ dPa·s). The latter will be helpful during the discussion of the results.

The laser used in this study was a commercial Yb-doped fiber amplifier femtosecond laser (Satsuma, Amplitude Systèmes Ltd. Pessac, France.), and the experimental conditions of laser inscriptions were the following: laser operating at 1030 nm, 250 fs delivering pulses, and 100 kHz repetition rate. The laser beam was focused at a depth of 200 μm using a 0.6 NA aspheric lens (estimated beam waist *w* ~1.5 μm) with the incident beam set perpendicular to the surface of the sample. The samples are affixed to a translation stage, which can be moved in the three dimensions of space, and the direction of the linear polarization was controlled using a λ/2 waveplate mounted on a rotation stage.

**Figure 1:**
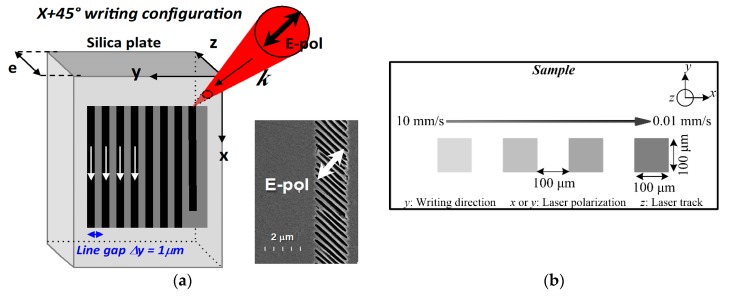
(**a**) Experimental setup scheme for X + 45° writing configuration and SEM image of sub-wavelength periodic structure formed along a single irradiated line. Original image is courtesy of Dr. Cyril Hnatovsky. (**b**) Experimental laser writing design.

To understand how glass composition influences laser-induced modifications and thermal stability, a series of squares were inscribed with a femtosecond laser in glass samples mentioned above. For each inscribed square, the laser writing direction is set to be the X axis, and the laser light polarization is oriented either parallel (Xx configuration, //) or perpendicular (Xy configuration, ⊥) to it. Then, using a scanning speed *v*, we created several squares 0.1 × 0.1 mm^2^, made up of a set of lines with a line spacing Δ*y* = 1 μm to have a uniform anisotropic area and avoiding any diffraction effects. Scheme about the laser writing procedure is shown in Figure 1 and more details can be found in Reference [23].

As pulse energy used during laser irradiation is set to be above the fs-*Type II* modification threshold (T2), the index change of irradiated regions is highly anisotropic, and its magnitude can be as large as 10^−2^ [16]. Such index change demonstrates thermal stability at 1000 °C for a few hours [9], which is a critical advantage for its high temperature application. According to previous research, fs-*Type II* modifications of SiO_2_ (Suprasil CG^®^) happen above an energy threshold of 0.4 μJ for laser polarization parallel to the laser scanning (λ = 1030 nm, Δt_p_ = 300 fs, RR = 100 kHz, and NA = 0.6) [24]. Therefore, in order to study the writing kinetic of *type-II* modifications, we performed the experiments by varying pulse energy from 0.3 μJ to 4 μJ. In order to study the impact of the writing speed on thermal stability, the scanning speed *v* was adjusted from 0.1 to 10 mm/s.

After femtosecond laser irradiation, the amount of optical retardance (*R*), induced by laser irradiation, was measured with an Olympus BX51 polarizing optical microscope equipped with a “De Sénarmont” compensator. The compensator couples a high precision quarter waveplate (made of quartz) with a 180-degree rotating analyzer to provide retardation measurements, yielding an accuracy of retardation measurements that approaches one thousandth of the wavelength [25]. The retardance is defined as *R* = *B* × *l*, where *B* is the linear birefringence (typ. n_e_-n_o_) and *l* is the thickness of the birefringent layer inscribed by the laser (and often corresponds to the laser track length expressed in nm). Hence, when a laser irradiates a sample and a birefringent object is formed, the measured *R* value corresponds to the amount of *B* integrated over the laser track length (in the z direction from Figure 1).

Moreover, the sample cross sections were investigated using a Scanning Electron Microscope (FEG-SEM, Field-Emission Gun Scanning Electron Microscope, ZEISS SUPRA 55 VP, 1 kV accelerating voltage, Oberkochen, Germany) after cleaving the samples using a diamond pen.

To investigate the thermal stability of the written fs-*Type II* modifications, the samples were isochronally (*δt* = 30 min step) heat treated with an incremental increase (typ. *ΔT* = 50 °C) of temperature T up to 1400 °C. After each annealing step, retardance *R* of the squares was measured at room temperature after natural cooling and normalized with respect to the retardance value from the as-irradiated glass.

## 3. Results

### 3.1. Impact of the Writing Speed

**Figure 2:**
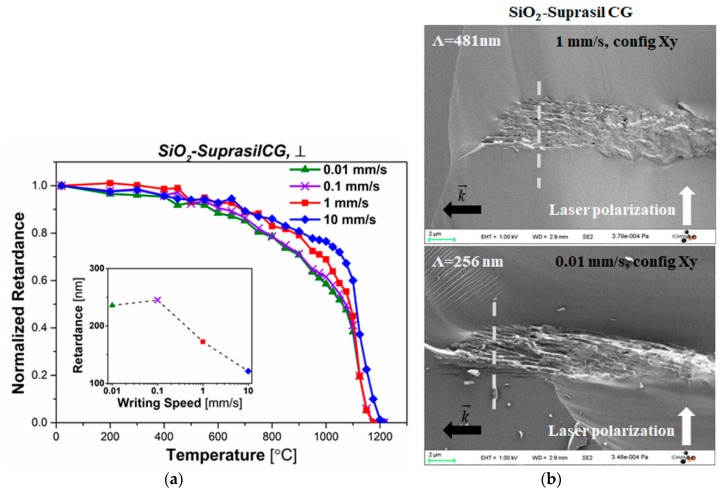
(**a**) Normalized retardance (*R_norm_*) as a function of temperature for SiO_2_ (Suprasil CG^®^) samples inscribed by different writing speed (0.01 mm/s, 0.1 mm/s, 1 mm/s, and 10 mm/s). The other laser parameters are: 2 μJ/pulse, 1030 nm, 250 fs, 100 kHz, NA = 0.6, laser polarization ⊥ to the writing direction. Measurements were performed at room temperature. **Inset**: initial retardance, measured before any annealing, according to the writing speed. (**b**) FEG-SEM, Secondary electron micrographs of the cross section of laser tracks for *v =* 1 mm/s (upper) and 0.01 mm/s (lower speed).

Figure 2a displays the normalized retardance (*R_norm_*) as a function of isochronal annealing temperature for a SiO_2_ sample from Heraeus (Suprasil CG^®^), with different writing speeds, namely: 0.01 mm/s, 0.1 mm/s, 1 mm/s, and 10 mm/s. In this experiment, the pulse energy was set to 2.0 µJ/pulse. In addition, retardance values as a function of writing speed for the non-heat-treated samples were measured and are reported in the inset graph of Figure 2a. It shows that with the higher writing speed, the lower retardance value would be achieved, and agrees well with the tendency reported in Reference [26]. From Figure 2a, some common features can be found. First, there is a slight decrease in the normalized retardance value from 400 °C to 600 °C corresponding approximately to 10% decay of the retardance strength. This feature might be attributed, according to the literature, to the erasure of point defects centers (E’, ODC, NBOHC) [27]. In the 800–1000 °C temperature range, the faster decay of normalized retardance was found for low writing speeds (0.01 mm/s and 0.1 mm/s). This decline might be related to the erasure of Si-O three and four-fold rings at 800–900 °C [4,27] related to the relaxation of glass densification (*Type I* contribution) or of the strong compressive stress. Finally, for annealing temperature above 1000 °C, a very steep decrease of the birefringence is observed. *R_norm_(T)* reaches zero at ~1175 °C for all samples.

Writing speed *v* is a key factor for formation of nanogratings, as it directly relates to the overlap between two subsequent pulses. In general, it can be estimated by the formula 1-v/(f*D), where *v* is the writing speed, *f* is the repetition rate and *D* is the laser beam diameter [26]. According to previous results, a decrease in writing speed yields to a higher overlap rate, which further creates a higher number of nanogratings with shorter average spacing [28]. The results shown in Figure 2b are in agreement with these results. Indeed, the spacing of nanogratings (Λ) is decreased from ~481 ± 10 nm to ~256 ± 10 nm as the writing speed *v* is decreased from 1 mm/s to 0.01 mm/s. At the same time, by comparing the thermal behavior of all samples with different writing speeds in Figure 2a, the sample inscribed by fast writing speed (*v* = 10 mm/s) demonstrates the highest thermal stability, accompanied with a sharp decay at 1100 °C. It completely annealed out after 30 min at ~1210 °C. This temperature is slightly higher than other samples inscribed at lower writing speeds. However, in view of Figure 2a,b, whether there is a correlation between thermal stability of fs-*Type II* nanogratings and nanogratings spacing still need further investigations and in particular a thorough analysis on the impact of nanolayers porosity (filling factor, core size). These results are shortly commented in the last section of the discussion.

### 3.2. Impact of Cl and OH Impurities

**Figure 3:**
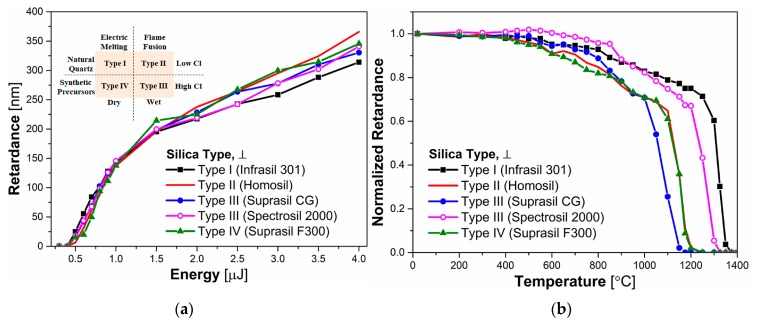
(**a**) Retardance as a function of pulse energy for SiO_2_ glasses: Type I (Infrasil 301^®^), II (Homosil^®^), III (Suprasil CG^®^ and Spectrosil 2000^®^), and IV (Suprasil F300^®^). Laser parameters are: 1030 nm, 250 fs, 100 kHz, 100 µm/s, NA = 0.6, laser polarization ⊥ to writing direction. Inset: category and characteristics of sample; (**b**) Normalized retardance (*R_norm_*) as a function of temperature for all SiO_2_ glasses. Pulse energy is 2 μJ/pulse and other laser conditions are same mentioned above. The measurements were performed at room temperature.

To reveal the impact of Cl and OH impurities on writing kinetics of fs-*Type II* modifications and their thermal behavior, five silica glass samples fabricated by different processes were studied. Based on the classification proposed by Brückner in 1970 [29], we categorized samples into four types as shown in the inset of Figure 3a: Type I silica is fused from natural quartz by electrical melting and its commercial name is Infrasil 301^®^ (OH < 8 ppm; Cl < 0.15 ppm). Type II silica is fused from natural quartz by plasma flame fusion and contains 380 ppm OH and <1 ppm Cl. Type III (Suprasil CG^®^; Spectrosil 2000^®^) and Type IV silica (Suprasil F300^®^) were synthetic and were prepared respectively, by electrical melting, or plasma-activated chemical vapor deposition (PCVD). The main similar point between types III and IV silica is the Cl content, on the order of 100 ppm [29], while the most significant difference is the OH content: which is 400–1000 pm in the former (wet sample) and <3 ppm in the later (dry sample) [30]. In addition, due to different raw materials used for silica glass production, that is natural quartz for Type I and II silica and synthetic precursors for Type III and IV silica, the latter are expected to be purer than the formers in terms of trace impurities, such as Al, etc. while containing higher level of Cl. Again, impurity contents for the various silica types are summarized in Table 1.

Figure 3a shows the retardance values measured for all investigated silica glasses, manufactured by the different techniques explained above, as a function of the laser pulse energy. The first interesting result is that there was no variation of the T2 threshold (0.4 μJ/pulse) with respect to the glass manufacturing technique used. Each sample exhibited a similar trend in their writing kinetics (i.e., retardance versus pulse energy). In all cases the retardance *R* was found to increase sharply from 0 nm to 200 nm in the 0.4–1.5 μJ/pulse energy domain. However, for higher pulse energies, up to 4 μJ/pulse the retardance increased more slowly. From the results of Figure 3a, it can be stated that the origin of increasing R for all samples is similar, i.e., the formation of a periodic array of porous nanoplanes isolated by non-porous glass [4,7], cooperating to form birefringence [4,31]. Furthermore, it is also reasonable to conclude that the impact of fabrication techniques and impurities content on the writing kinetics of fs-*Type II* modifications for all silica samples is not significant, which agrees well with results reported in Reference [16].

Figure 3b displays the evolution of the normalized retardance (*R_norm_*) of all silica glasses as a function of the annealing temperature. In this Figure, it can be found that retardance values for all samples started to decay at 400 °C. After the step at which the silica samples were kept at 1025 °C for 30 min, the decay of normalized retardance was ~32% for Types II, III (Suprasil CG^®^), and IV silica, while only ~19% for Types I and III (Spectrosil 2000^®^) silica. After this step, a sudden drop of retardance is observed. For type III silica (Suprasil CG^®^), which is the sample to exhibit this feature at the lowest temperature, no retardance could be detected after 1175 °C. For Type II and IV silica, normalized retardance started to diminish drastically at 1075 °C and became hard to measure at 1225 °C, which was 50 °C higher than Type III. Compared with Type II, III (Suprasil CG^®^) and IV, Type I and III (Spectrosil 2000^®^) glass exhibited a better thermal stability and a similar decreasing trend. The normalized retardance kept decreasing slowly to ~75% until ~1300 °C and there is a very sharp erasure process after that. Finally, the normalized retardance of Type III (Spectrosil 2000^®^) and I silica could not be detected after 1320 °C and 1400 °C respectively, indicating that the fs-*Type II* modifications of these two samples were completely erased. It is worth pointing out that these values are beyond the estimated glass transition temperature of 1260 °C for Type I fused quartz with low OH and aluminum contents [32].

Now, from Figure 3b and by looking at the evolution of *R_norm_* as a function of temperature, some typical characteristics can be revealed. At the same OH levels, by comparing Type I (Infrasil 301^®^, Cl < 0.15 ppm) with Type IV (Suprasil F300^®^, Cl ~2500 ppm) silica or Type II (Homosil^®^, Cl < 1 ppm) with Type III (Spectrosil 2000^®^, Cl < 0.15 ppm; Suprasil CG^®^, Cl ~2500 ppm) silica, we can find that silica glasses produced by less pure natural quartz with the lower level of Cl all demonstrate the better thermal stability than the ones made from synthetics precursor. This indicates that high level of Cl would be one factor to deteriorate the overall thermal stability of fs-*Type II* modifications. Similarly, by comparing Type I (Infrasil 301^®^, OH < 8 ppm) with Type II (Homosil^®^, OH ~380 ppm) silica or Type IV (Suprasil F300^®^, OH ~0.1 ppm) with Type III (Suprasil CG^®^, OH ~830 ppm) silica, it is found that dry glasses demonstrate a higher thermal stability than wet glasses when produced by the same precursor material. From these observations, one first conclusion to be drawn is that fs-*Type II* modifications in dry silica glass with lower levels of Cl, such as Type I silica (Infrasil^®^) in this study, would exhibit a better thermal stability. We note also that Cl has a stronger impact than OH (for a fixed concentration) on the thermal stability.

### 3.3. Influence of GeO_2_ Content

To study the influence of GeO_2_ content on writing kinetics and thermal stability of fs-*Type II* modifications, several *x* GeO_2_- (1-*x*) SiO_2_ glasses with various GeO_2_ concentrations ranging from 1.9 mole% to 16.9 mole% were studied in this work. This concentration range is typical for standard optical fibers from single mode fibers like SMF28 (typ. 4 mole% GeO_2_) to multimode fibers. These samples are optical fiber preforms. They were all manufactured by plasma chemical vapor deposition (PCVD) with low OH contents (<0.1 ppm) and high level of Cl (~2000 ppm).

**Figure 4:**
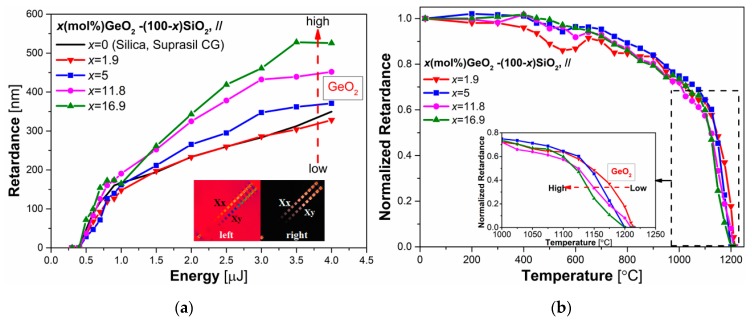
(**a**) Retardance as a function of pulse energy for *x* GeO_2_-(1-*x*) SiO_2_ glasses (*x* = 1.9 to 16.9 mole%). Inset graph of the sample 16.9 GeO_2_-83.1 SiO_2_ glass: **left:** optical microscope (bottom light illumination) cross a full-wave plate, polarizer, and analyzer; **right:** optical microscope (bottom light illumination) cross polarizer and analyzer; (**b**) Normalized retardance (*R_norm_*) as a function of temperature for *x* GeO_2_-(1-*x*) SiO_2_ glasses (*x* = 1.9 to 16.9 mole%). Pulse energy is 2 μJ/pulse. Laser parameters are: 1030 nm, 250 fs, 100 kHz, 100 µm/s, NA = 0.6, polarization // to writing direction.

Figure 4a displays the retardance values as a function of pulse energy for the four germanosilicate glasses investigated. Results of pure SiO_2_ (Suprasil CG^®^) is added to serve as a reference. Insets are two microscope images taken between crossed polarizers that reveal the formation of birefringence. The left inset was made using a full order waveplate and highlights the slow axis orientation which flips of 90° (from yellow to blue color) when the writing laser polarization was changes from x to y orientation. This signature of polarization dependent birefringence confirms indirectly the formation of nanogratings, which have been previously confirmed by SEM [17]. As it can be seen, the threshold T2 [7], related to formation of nanogratings with form birefringence, is about 0.4 µJ for all tested samples in our experimental conditions, which is the same value of the T2 threshold of pure silica samples shown in Figure 3a. It should also be noted that we did not observe any variation of T2 with the increasing content of GeO_2_ in the investigated range. From 0.4 µJ to 1 µJ, the evolution of *R* as a function of pulse energy for all samples nearly is identical. For higher pulse energies, the slower growth of retardance of all samples can be found. In addition, from 1 µJ to 4 µJ, it has been reported that overall magnitude of *x* GeO_2_-(1-*x*) SiO_2_ binary glasses is higher than retardance values of pure SiO_2_ sample due to a higher amount of glass decomposition within nanolayers. Such higher form birefringence is also observed in GeO_2_ glass [20] or GeO_2_ doped glasses [19] and is explained by smaller pore size of GeO_2_ but a higher decomposition level (resulting in a higher porosity filling factor) in SiO_2_-GeO_2_ [17] and thus stronger birefringence response [20]. However, opposite trend of writing kinetics has also been observed, showing that the higher the GeO_2_ content [17], the lower the retardance could be obtained when nanogratings were inscribed by the higher repetition rate (500 kHz), writing speed (0.5 mm/s), and higher pulse duration (300 fs). Further systematic study is needed to reveal the impact of writing parameters on fs-*Type II* modifications in germanosilicate glasses.

Figure 4b displays the normalized retardance *R_norm_* of germanosilicate glasses as a function of annealing temperature and shows some characteristic features. In this experiment, we also thermally treated samples written at 2.0 µJ/pulse, to induce fs-*Type II* modifications. As we can see, at the beginning, there is a slight increase of the retardance value from 600 °C to 800 °C, and it is more obvious for germanosilicate glasses with less GeO_2_ content. Similar behavior of the birefringence has been observed in SiO_2_ [9,24], 50SiO_2_-50Al_2_O_3_ pellet [24] and slightly Ge-doped optical fibers [8]. Briefly, it is assumed that this observed unexpected increase is related to some contribution of residual part of *Type I* (defects, densification, and related stress), which erased at low temperature, resulting in a higher birefringence response [24]. As the annealing temperature increases, the induced stress relaxes gradually, lessening the refractive index and further decreasing retardance. Finally, above 1100 °C, retardance diminishes dramatically, and no birefringence can be found after ~1200°C annealing. From the inset of Figure 4b, it should be also noted that the “erasing slope” is inversely dependent on the GeO_2_ concentration and increases from 1150 °C to 1200 °C as GeO_2_ decreases from 16.9 mole% to 1.9 mole%. Such result matches with the estimated glass transition temperature (T_g_) for germanosilicate glasses fabricated by Sol-gel technique with different GeO_2_ content [33]. In addition, it is also generally accepted that viscosity of glass is reduced by the high level of OH concentration, which further reduces the glass transition temperature (T_g_) [33]. Therefore, due to the very fact that samples in our case were fabricated with less OH content and the fact that germanosilicate glasses are composed of an interpenetrated and interconnected structure of SiO_4_ and GeO_4_ tetrahedral units [34], viscosity of glass is expected to change slowly as the concentration of GeO_2_ varies from 1.9 mole% to 16.9 mole%. This is confirmed by our viscosity measurements made in two of these samples shown in Figure 4b (red and blue curves). It explained why incorporating GeO_2_ has less impact on overall thermal behavior of all germanosilicate glasses in comparison with pure silica glasses (especially, Type II and IV silica) presented Figure 3b.

### 3.4. Impact of Dopants

In this part, the impact of dopants on thermal stabilities of fs-*Type II* modifications is discussed. In addition to the thermal behavior of the pure silica (Infrasil 301^®^) and the germanosilicate glasses presented in this work, results of titania-silicate glass (ULE^®^; 7 mole% TiO_2_) and aluminoborosilicate (Borofloat33^®^; 81 mole% SiO_2_, 13 mole% B_2_O_3_, 4 mole% Na_2_O/K_2_O, 2 mole% Al_2_O_3_) are also collected and compared.

**Figure 5:**
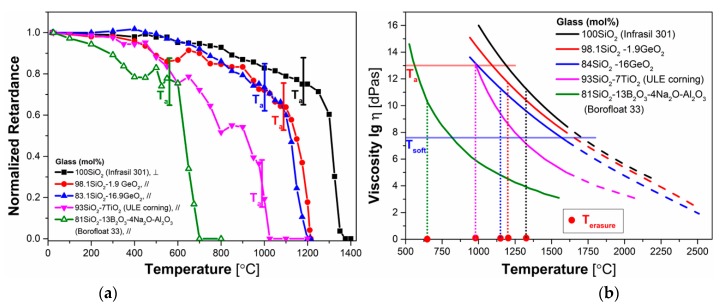
(**a**) Normalized retardance (*R_norm_*) as a function of annealing temperature *T* for SiO_2_ (Infrasil 301^®^), 1.9GeO_2_-98.1SiO_2_, 16.9GeO_2_-83.1SiO_2_, 93SiO_2_-7TiO_2_ (ULE^®^), and 81SiO_2_-13B_2_O_3_-4Na_2_O/K_2_O-2Al_2_O_3_ (Borofloat33^®^). (**b**) Viscosities of glasses with different compositions as a function of temperature (dash line: extrapolated part). Annealing temperature (T_a_) is defined when log(η) = 13 dPa·s. Softening temperature (T_soft_) is defined when log(η) = 7.6 dPa·s. Erasure temperature (T_erasure_) is defined at half-amplitude of the final erasure slope of *R_norm_(T)*.

Figure 5a shows the normalized retardance (*R_norm_*) as a function of temperature. The thermal stability was studied through an annealing experiment of isochronal (*δt* = 30 min) annealing steps (ΔT = 50 °C or 25 °C). Note that the thermal stability is related to a couple (t,T) and thus related to a thermal energy often called demarcation energy that is usually written as k_B_.T.ln(k_0_.T) for simple cases [35] where k_0_ is the pre-exponential factor in the Arrhenius rate constant of the erasure reaction and k_B_ is the Boltzmann constant. If you determine the k_0_ value using multiple isothermal or isochronal annealing experiments both isothermal and isochronal accelerated ageing are equivalent and bring similar information’s allowing also the possibility to perform reliable lifetime prediction. In our case, the curves in Figure 5a represents the “stability curve” provided that the criterion (*δt.*k_0_)^-(ΔT/Tmax)^ ≪ 1 is fulfilled [35] where k_0_ is the pre-exponential factor in the Arrhenius rate constant of the erasure reaction. For nanogratings written in silica, k_0_ has been estimated to be around de 5 × 10^5^ – 5 × 10^7^ s^−1^ [36] depending on the laser writing parameters. So, when this criterion is respected, each point can be considered independent of each other. If the criterion is not fulfilled this means that our measurements are slightly under-estimating the real thermal stability. 

As can be seen from Figure 5a, *R_norm_* of ULE^®^ and Borofloat33^®^ first decreases smoothly until 600 °C, leading to a 20% decrease for both two samples. At higher temperatures, the decrease in *R_norm_*(T) is accelerated and neither retardance nor refractive index contrast can be found at 650 °C for Borofloat33^®^ and at 1025 °C for ULE^®^, respectively. The studies of mechanisms leading to decay in retardance at high temperature is exemplified in Reference [24] and is attributed to the erasure of point defects below 500 °C. Then there are at least two additional contributions: the first one (intermediate temperature range, typ. 0.8T_a_ to T_a_) is related to the stress-induced birefringence generated inside and outside the nanolayers; the second one (sharp final decay above T_a_) is related to the nanotexturation itself (porosity and periodicity) leading to form birefringence. For germanosilicate glasses, the sharp decay process starts to take place at 1100 °C and retardance cannot be measured at ~1200 °C, showing a better thermal stability than ULE^®^ and Borofloat33^®^. For pure silica (Infrasil 301^®^), from ~1300 °C and above, a steep decrease of *R_norm_* occurs up to 1400 °C for 30 min. To sum up, thermal stability of fs-*Type II* modifications on the various samples can be classified as follow (from higher to lower thermal stability): 100 SiO_2_ > 1.9 GeO_2_ - 98.1 SiO_2_ > 16.9 GeO_2_-83.1 SiO_2_ > 93 SiO_2_-7 TiO_2_ > 81 SiO_2_-13 B_2_O_3_-4 Na_2_O/K_2_O-2 Al_2_O_3_.

Viscosities η of glasses with dopants were plotted as a function of temperature (Figure 5b). The viscosities of Infrasil 301^®^, ULE^®^, and Borofloat33^®^ were obtained from the commercial data. The viscosity of germanosilicate glasses was measured and extrapolated above 1500 °C. The annealing temperature (T_a_) of the glasses, related to the glass structural relaxation, were determined from the curves when the log(η) reaches 13 dPa·s. From the Figure 5b, it is evident that viscosities η(T) of all the samples decrease when temperature increases. In addition, it is clear that pure silica (Infrasil 301^®^) has the highest viscosities and T_a_ (1180 °C) compared with other silicate glasses. Furthermore, compared with pure silica, the viscosity and T_a_ of GeO_2_-doped glass samples only slightly decreases with the increase of GeO_2_ content, indicating that there are minor changes of the glass network structure after doping with GeO_2_. However, when it comes to titania-silicate glass and aluminoborosilicate, the viscosities and T_a_ decreases drastically, showing that high doping levels of titania oxide (TiO_2_) or boron trioxide (B_2_O_3_) would significantly modify the initial silica glass network structure. Combined with the results shown in Figure 5a, we found that a steep decay always starts to take place after T_a_, typ. around T_erasure_ (reported in Figure 5b), and is always fully erased for a temperature slightly lower than T_soft_. So, the thermal stability of fs-*Type II* modifications seems to be correlated qualitatively but not quantitatively to their T_a_. A more accurate view might be related to the temperature corresponding to a glass structural relaxation time τ(T) of 30 min, usually defined by τ(T) = η(T)/G(T), with G being the glass shear modulus.

## 4. Discussion

In agreement with Reference [37], fs-*Type II* modifications or nanogratings formation in silica or glass is limited by internal structural relaxation of glass when using high pulse energy or high repetition rate. In our studies, the thermal stability or nanogratings erasure, also might be related to the glass structural relaxation τ(T) but this is likely more complex due to the multiple steps’ erasure mechanism. Indeed, the nanogratings erasure relates to oxide decomposition/formation in SiO_2_, nanoscale phase separation in other glasses, oxygen chemical migration/reaction, all taking time. However, they do not depend only on the viscosity e.g., the viscous flow but also mostly the surface tension, are especially important for voids and pores erasure. Especially, at high temperature, chemical migration coefficient and viscosity are strongly interconnected with each other. It explains why “thermal stability” of fs-*Type II* modifications in silicate glasses is in the same order as their annealing temperature (T_a_): the higher T_a_, the most stable behavior at high temperature of fs-*Type II* modifications can be found and expected.

It has been shown that doping titania oxide (TiO_2_) or boron trioxide (B_2_O_3_) into silicate glasses would significantly affect the silicate network structure. For instance, complex silicate sheets can be depolymerized into simpler silicate structures by doping with TiO_2_ [38] and can yield to weak B-O bonds with low bond energies when the glass is doped with B_2_O_3_ [39]. As a result, viscosity and T_a_ decrease, making fs-*Type II* modifications easier to be erased at high temperature as shown in Figure 5a. In addition to that, impurities, such as hydroxyl (OH) and chloride (Cl), are also proven to be another factor influencing the thermal stability of fs-*Type II* modifications as we shown in Section 3.2. For OH impurities, it has generally been agreed that water incorporated in glass structure is in the form of hydroxyl ions (SiOH) and the presence of SiOH in glasses therefore increases the concentration of non-bridging anions, which further decrease the viscosity of glass and thus T_a_ [40]. It explains why fs-*Type II* modifications in dry glasses (Type I and IV silica) in our study shows better thermal performance than wet glasses (Type II and III silica). Similarly, Cl as another impurity that relies to the manufacturing process would decrease the viscosity, T_a_ and relaxation time of the glass [41], making fs-*Type II* modifications in Type III and IV silica in our study become less stable.

When it comes to the influence of germanium dioxide (GeO_2_) in Figure 4b, we can see that by doping with GeO_2_ up to approximately 17 mole%, the thermal behavior of fs-*Type II* modifications is somewhat similar. It is attributed to the fact that GeO_2_ and SiO_2_, when mixed, form a continuous random mixture, and the two networks are strongly connected or "interpenetrated" [34]. That is why adding a few mole% of GeO_2_ does not change so much viscosity, T_a_ and thus thermal stability. However, we also observed, at high temperature, the steep decay to be weakly dependent of GeO_2_ concentration (inset of Figure 4b) in agreement with our viscosity measurements.

For the impact of writing speed on thermal stability of fs-*Type II* modifications, it is not easy to draw a conclusion at this step. For example, we cannot say that higher nanogratings spacing induced by slow writing would lead to a better thermal performance of fs-*Type II* modifications, because of some counter-examples. From the perspective of porosity erasure, at the same level of viscosity and by the same annealing procedures, it would take more time to erase the larger pores rather than the small ones. However, this may not directly be related to porosity or periodicity. It is true that we observed a large difference in thermal stability in the 900–1100 °C temperature range from Figure 2a, but this difference reduces when the erasure is total, around 1200 °C. This significant difference in retardance we observed might be rather related to stress effects, contributing to our retardance measurements (stress-induced birefringence), which usually relax during a thermal treatment at 0.8–0.9.T_a_. Based on Reference [42], higher deposited energy dose (i.e., lower speed) leads to higher cumulative stress: each nanolayer undergoes a net volume expansion [43], which results in some residual compressive stress. Consequently, slow writing speed resulting in more nanolayers per unit of length (i.e., shorter periods) would yield to higher cumulative residual stress and thus stress birefringence values. So following this view, from Figure 2a, we can estimate that low speed writing leads to a high stress induced birefringence, contributing to 40% of the total retardance, instead of 20% for high writing speed at ~1100 °C.

## 5. Conclusions

In this work, we mainly investigated the impacts of writing speed, impurities (Cl and OH), and GeO_2_, TiO_2_ dopants on thermal stability of *Type II* modifications and their writing kinetics by IR femtosecond laser in silica-based glasses. From this study, we found that the impact of writing speed on *fs-Type II* modifications thermal stability is not intuitive in pure silica. High level of OH and Cl impurities would reduce the *Type II* modifications thermal stability, while causing negligible impact on their writing kinetics. We observed that by adding GeO_2_ into SiO_2_ up to ~17 mole%, the thermal stability is only slightly influenced, while the magnitude of the retardance is found to be dependent on GeO_2_ concentration. By comparing the thermal behavior and viscosity of Borofloat33^®^, ULE^®^, GeO_2_-SiO_2_ and SiO_2_ (Infrasil 301^®^) glasses, we found that the thermal stability of *Type II* modifications is partly related to the glass structural relaxation or viscosity but not solely due to additional mechanisms involving nanopores erasure, oxide decomposition/formation, and chemical migration. It also explains the variation of thermal behavior induced by GeO_2_ and impurities. These results provide a reference work for writing such “*Type II* fs-IR” modifications on multicomponent silicate fibers to make fiber Bragg gratings with high thermal stability.

## Figures and Tables

**Table 1:** Designations, compositions, impurities^1^, and T_a_^2^ of samples for each section.

Section	Designations	Compositions(mole%)	Impurities^1^ (ppm)		T_a_^2^ (°C)
			OH	Cl	
**3.1** **Speed**	Suprasil CG^®^	100 SiO_2_	830	< 2500	1100
**3.2** **OH; Cl**	Type I (Infrasil 301^®^)	100 SiO_2_	8	< 0.15	1180
	Type II (Homosil®)		380	< 1	1180
	Type III (Suprasil CG^®^)		830	< 2500	1100
	Type III (Spectrosil 2000^®^)		800–1200	< 0.15	1025
	Type IV (Suprasil F300^®^)		0.1	2500	1110
**3.3** **GeO_2_**	Germanosilicate	*x*GeO_2_-(1-*x*) SiO_2_, *x* = 1.9, 5, 11.8, 16.9	< 0.1	~2000	990–1090
**3.4** **Dopants**	Infrasil 301^®^	100 SiO_2_	8	< 0.15	1180
	Germanosilicate	1.9 GeO_2_-98.1 SiO_2_	< 0.1	~2000	1090
	Germanosilicate	16.9 GeO_2_-83.1 SiO_2_	< 0.1	~2000	990
	ULE corning^®^	7 TiO_2_-93 SiO_2_	-----	-----	900
	Borofloat33^®^	81 SiO_2_-13 B_2_O_3_-4 Na_2_O/K_2_O -2 Al_2_O_3_	-----	-----	663.6

^1^ Impurities: Impurity concentrations were obtained from the manufacturers, Fourier transform infrared spectroscopy (FTIR), or electron probe micro-analysis (EPMA). ^2^ T_a_: Annealing temperature of samples was obtained from the manufacturers or from measurements. T_a_ is defined as the temperature at which the viscosity is 10^13^ dPa·s.
